# Medical Advice for Sick-reported Students (MASS) in intermediate vocational education schools: design of a controlled before-and-after study

**DOI:** 10.1186/s12889-017-4530-2

**Published:** 2017-06-29

**Authors:** Madelon K Van der Vlis, Marjolein Lugtenberg, Yvonne T.M. Vanneste, Wenda Berends, Wico Mulder, Rienke Bannink, Amy Van Grieken, Hein Raat, Marlou L.A. de Kroon

**Affiliations:** 1 Department of Public Health, Erasmus University Medical Centre, PO Box 2040, 3000 CA Rotterdam, The Netherlands; 2 Department of Youth Health Care, Regional Public Health Service West Brabant, PO Box 3024, 5033 DA Tilburg, The Netherlands; 3 Physicians Association Youth Health Care The Netherlands, Churchilllaan 11, 3527 GV Utrecht, The Netherlands; 4 Department of Youth Health Care, Regional Public Health Service Amsterdam, PO Box 2200, 1000 CE Amsterdam, The Netherlands; 5 Department of Youth Health Care, Regional Public Health Service Rijnmond, PO Box 3074, 3003 AB Rotterdam, The Netherlands

**Keywords:** Adolescent, Health, Youth Health Care, School absenteeism, MASS intervention, Public health

## Abstract

**Background:**

School absenteeism, including medical absenteeism, is associated with early school dropout and may result in physical, mental, social and work-related problems in later life. Especially at intermediate vocational education schools, high rates of medical absenteeism are found. In 2012 the Dutch intervention ‘Medical Advice for Sick-reported Students’ (MASS), previously developed for pre-vocational secondary education, was adjusted for intermediate vocational education schools. The aim of the study outlined in this paper is to evaluate the effectiveness of the MASS intervention at intermediate vocational education schools in terms of reducing students’ medical absenteeism and early dropping out of school. Additionally, the extent to which biopsychosocial and other factors moderate the effectiveness of the intervention will be assessed.

**Methods:**

A controlled before-and-after study will be conducted within Intermediate Vocational Education schools. Schools are allocated to be an intervention or control school based on whether the schools have implemented the MASS intervention (intervention schools) or not (control schools). Intervention schools apply the MASS intervention consisting of active support for students with medical absenteeism provided by the school including a consultation with the Youth Health Care (YHC) professional if needed. Control schools provide care as usual. Data will be collected by questionnaires among students in both groups meeting the criteria for extensive medical absenteeism (i.e. ‘reported sick four times in 12 school weeks or for more than six consecutive school days’ at baseline and at 6 months follow-up). Additionally, in the intervention group a questionnaire is completed after each consultation with a YHC professional, by both the student and the YHC professional. Primary outcome measures are duration and cumulative incidence of absenteeism and academic performances. Secondary outcome measures are biopsychosocial outcomes of the students.

**Discussion:**

It is hypothesized that implementing the MASS intervention including a referral to a YHC professional on indication, will result in a lower level of medical absenteeism and a lower level of school drop outs among intermediate vocational education students compared to students receiving usual care. The study will provide insight in the effectiveness of the intervention as well as in factors moderating the intervention’s effectiveness.

**Trial registration:**

Nederlands Trial Register NTR5556. Date of clinical trial registration: 29-Oct-2015.

## Background

Obtaining a basic educational degree increases the chances of finding a permanent skilled job and is associated to better health. Students who break off their education prematurely often find themselves in a more vulnerable situation with regard to developing illnesses, being unemployed and being involved in criminal activities, as compared to students who hold a basic educational degree. Reasons to drop out of school prematurely, as reported by students, vary from learning and motivation difficulties, behavioural, (mental) health problems to problems at home [1–5].

School absenteeism is often a precursor of school dropout and absenteeism in future careers [4, 6–10]. It can be divided into unexcused (truancy) and excused absenteeism. The most common form of excused absenteeism is absence due to sickness reporting or sick leave, so called medical absenteeism. Medical absenteeism is perceived twice as often as truancy among adolescent students [11]. Particularly at intermediate vocational educational schools, high rates of medical absenteeism and school dropout can be found. Of all students entering intermediate vocational education, only 50% passes on to the second year and after five years approximately 25% leaves school without a degree [12]. Investing in effective methods regarding the prevention of extensive medical absenteeism at intermediate vocational education schools is therefore relevant.

The Dutch Youth Health Care (YHC) system offers preventive health care for children and adolescents between 0 and 18 years old. It aims to foster the optimal trajectory for growth and development in children and to provide anticipatory guidance to prevent negative outcomes later in life [13]. In general, the YHC system does not focus actively on students with extensive medical absenteeism. The current policy of most intermediate vocational education schools does not include an active identification of students with extensive absenteeism and referrals to YHC professionals are limited [14, 15]. Because of the high rate of medical absenteeism at intermediate vocational education schools and the complex underlying problems, however, the YHC system could play an important role in addressing medical absenteeism [16–20].

In 2012, the Medical Advice for Sick-reported Students (MASS) intervention was developed [19] to address medical absenteeism more actively within students attending pre-vocational secondary education, the school level preceding intermediate vocational education. The aim of the MASS intervention is to limit medical absenteeism by arranging appropriate care, educational adjustments and adequate support for students [21]. The intervention focuses on the factors associated with extensive medical absenteeism: somatic, mental and physical complains [9, 22–25], poor lifestyle [11] and psychological, family and social problems of sick-reported students [9]. The schools and the YHC professionals collaborate directly within the MASS intervention to design and monitor a management plan that aims to optimize students’ health and maximize students’ participation in school activities [19, 25] In an effect study Vanneste et al. found a reduction of 15.1% in medical absenteeism among pre-vocational secondary education school students after implementing the MASS intervention [19].

As students in pre-vocational secondary education schools often continue their education at intermediate vocational education schools, medical absenteeism might be associated with the same determinants in both groups of students. Despite potential similarities between intermediate vocational education and pre-vocational secondary education students, there are also clear differences that need to be taken into account in designing interventions. Students in intermediate vocational education schools, for instance, are generally aged between 15 and 25 years old. Furthermore, the level of medical absence is often larger at intermediate vocational education schools and its nature is often more complex due to underlying biopsychosocial factors and financial problems. As a result, the MASS intervention has been adapted for intermediate vocational education schools and is currently being applied at several schools in the Netherlands [26–28]. However, evidence for its effectiveness is thus far lacking.

The aim of this study is to evaluate the effectiveness of the MASS intervention at intermediate vocational education schools, with a focus on the efforts of the YHC professional, in terms of reducing students’ medical absenteeism and early dropping out of school. In addition, we will assess to what extent biopsychosocial and other factors may influence the effect of the intervention.

## Methods/Design

### Study design

A controlled before-and-after study with a follow-up period of 6 months will be conducted. Students with extensive medical absence attending schools in which the MASS intervention has been implemented (intervention group) are compared to students attending schools in which the MASS intervention has not been implemented (control group).

The Medical Ethical Committee of Erasmus University Medical Centre Rotterdam has declared that the Medical Research Involving Human Subjects Act (also known by its Dutch abbreviation WMO) does not apply to this research protocol and issued a declaration of no objection (i.e. formal waver) for this study (MEC-2015-614). Therefore, the study can be carried out without further approval by an accredited research ethics committee.

### Setting

This study will be conducted in the Dutch setting of intermediate vocational education schools and will focus on students aged between 16 and 25 years. In the Netherlands, students under the age of 18 leaving lower secondary school have to continue a higher general secondary education or enter one of the five intermediate vocational education programs: technology, economics, agricultural, personal/social services or health care. The intermediate vocational education schools prepare students for a specialized trade by developing a particular expertise. After graduating from an intermediate vocational education school one can enter higher vocational education or start working [29].

### Participants

#### Youth health care organizations, schools and youth health care professionals

This study will be conducted in the Dutch regions of Amsterdam, Utrecht, West Brabant and Rotterdam. School recruitment will be organized in collaboration with three YHC organizations associated to the regions of Amsterdam, Utrecht and West Brabant. The YHC organization in Rotterdam will not be involved in the study as schools in this area have not yet implemented the MASS intervention and will therefore be allocated to the control group.

YHC organizations invite the intermediate vocational education schools to participate in the study. Schools that agree to participate will subsequently be visited by the researchers. It is expected that a total of 12 schools will participate. The allocation of the schools will be based on their current policy. Schools will be classified as an intervention school (IS) or as a control school (CS) based on their current use of the MASS intervention.

All YHC professionals of participating intervention schools will be offered a training in the MASS intervention at the start of the study.

#### Students

**Fig. 1:**
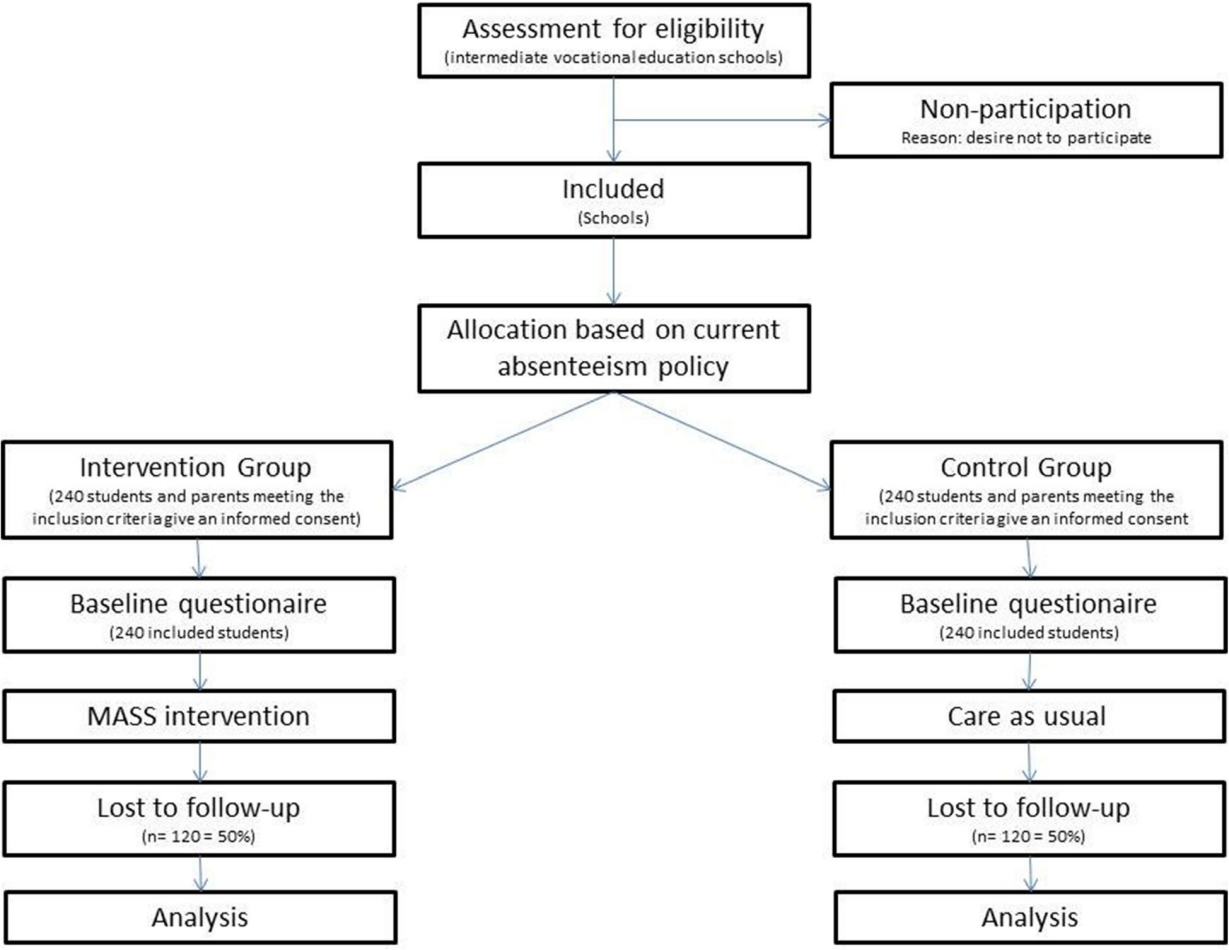
Flow chart of the MASS study

Students of participating schools are included in the study if they meet the criteria for extensive medical absenteeism, which is based on the MASS intervention at pre-vocational secondary education schools (i.e. reported sick for at least four times in 12 weeks or reported sick for more than six consecutive school days). We expect to include a total number of 480 students in the study with an equal distribution between the control and intervention group (see Fig. 1).

### Procedure

Participating schools are visited by the Erasmus MC researchers to explain the procedure of the study. The schools (usually an appointed coordinator) select students meeting the inclusion criteria (i.e. reported sick for at least four times in 12 weeks or reported sick for more than six consecutive school days) and invite them to participate in the study. All students receive an information letter and leaflet with information about the study. They are asked to provide informed consent. Participants in the study are invited to complete the baseline questionnaire. The coordinator will send the filled-out questionnaires back to the researchers on a regular basis. For students aged younger than 18 years of age qualifying for participation, parents also receive an information letter and brochure about the study at home provided by school; these parents can object to participation in the study by their child. After 6 months, a follow-up questionnaire will be sent to the students. Students will receive either a hard-copy version, a digital version or both versions, depending on their preference.

Students in intervention schools who have a consultation with the YHC professional will be asked by the YHC professional to complete an additional short questionnaire after consultation. The YHC professionals themselves are also asked to fill out a short questionnaire after each consultation. All questionnaires are to be dropped in sealed envelopes in a drop box which will be located at an agreed upon location with the coordinator of the school. The coordinator will send the questionnaires back to the researchers on a regular basis.

Data will be processed in accordance with the agreed guidelines of the Dutch Data Protection Authority Intervention [30].

### Intervention

#### Intervention group: the MASS intervention

The MASS intervention designed for intermediate vocational education schools consists of five steps: an active approach by the school after the first sick report, a meeting between student and school, a YHC professional referral if needed, the actual YHC professional consultation, and the further monitoring of the medical absenteeism and reintegration of the student [31] (Table 1).

**Table 1:** Description of the key steps of the MASS intervention

Step	Description
1	The school contacts the student actively in case of medical absence and asks about the context of the sickness report and condition of the student.
2	The school organizes a meeting with the student (and parents if student is <18 years old) in case of extensive medical absenteeism (criteria predefined by each individual school, apart from the MASS criteria).
3	The school refers the student to a YHC professional if considered to be needed.
4	A YHC consultation between student and YHC professional is organized
5	The school is responsible for monitoring the medical absenteeism and school-related implementation of the reintegration plan, if created.

*MASS* Medical Advice for Sick-reported Students

*YHC professional* Youth health care professional

The intervention focuses at two levels; the school level and the individual level. The implementation of the MASS intervention at school level includes agreements on how to actively monitor student absence, approach students with extensive medical absence by personal contact, request a consultation with the YHC professional, and arrange a follow-up for each student in the Care Advisory Team (CAT). The CAT includes social workers, attendance officer, YHC professionals, teachers and a Youth Care Office representative.

At an individual level students with extensive medical absenteeism will be identified by school and referred to YHC if needed. In the consultation, the YHC professional focuses on somatic, psychological and social factors potentially underlying the medical absenteeism (Table 2). Along with the student, and the parents if the student is younger than 18 years of age, a reintegration plan is formulated. Whichever reintegration plan is chosen, the YHC professional is accountable for the referral, the aftercare, communication with other care professionals, communication with the adolescent and monitoring the procedure accurately. All YHC professionals are trained in performing the MASS protocol [31].

**Table 2:** Description of key elements of the YHC consultation

Element	Description
1	The YHC professional and the student conduct a problem analysis
2	The YHC professional analyses the underlying problems by use of the biopsychosocial model
3	The underlying causes of the medical absence are defined
4	The possibilities of preventing recurrence in the future are discussed
5	The possibilities for extra treatments by health care professionals or other supporting professionals are discussed
6	An action plan regarding reintegration will be created, if needed
7	The YHC professional is responsible for communicating, with the consent of the student, the reintegration plan to all other involved parties without violating his/her professional secrecy

*YHC professional* Youth health care professional

#### Control group: care as usual

The control group consists of students attending Intermediate Vocational Education schools that have not implemented the MASS intervention. They continue to provide care as usual using their current absenteeism policy. This generally consists of a referral to a YHC professional on request of the student, when accessible consultation of the CAT and referring to the school attendance officer when student is younger than 18 years old.

### Measurements

The following primary and secondary outcome measures were formulated:

Primary outcomes measures

➢ Duration of the medical absence in days within the last 12 weeks at T1 (in comparison with T0).

➢ Cumulative incidence of the medical absenteeism, measured during six months after determining the extensive medical absenteeism.

➢ Academic performances as measured by passing onto the next year or obtaining a basic educational qualification degree.

➢ The extent to which the chosen educational program matches the student’s interests.

Secondary outcome measures

➢ Biopsychosocial problems of intermediate vocational education students after determining the extensive medical absenteeism; it concerns medical, physical, emotional, and psychiatric problems (e.g. depression, anxiety, ADHD), housing, financial problems and contacts in criminal justice.

➢ Realised changes/adjustments in treatment policy by specialists and other involved healthcare professionals for students with depression, anxiety, ADHD and autism spectrum disorders.

Specific outcomes related to the care by YHC professionals.

➢ The extent to which the YHC professionals work in accordance with the MASS protocol assessed during consultation with the student.

➢ Perceived value of the MASS intervention according to the YHC professionals and the students.

#### Covariates

The covariates are chosen based on comparison of baseline characteristics of intervention and control students. Demographic information regarding the participating students is collected at baseline including ethnicity, SES score, attending school, educational level, and parenthood. Information on a number of known risk factors for school absenteeism are collected at baseline and during follow-up, including age, gender, smoking status, alcohol consumption (abstainer, up to >7 servings/week) and drugs abuse (abstainer, soft drugs, and hard drugs). Biopsychosocial problems concerning medical, physical, emotional, and psychiatric problems (e.g. depression, anxiety, ADHD), housing, financial problems and contacts in criminal justice are included as covariates. Level of physical activity is assessed at baseline and follow-up.

### Data collection

Data collection takes place during 1,5 academic school years. All students complete a questionnaire at baseline (T0) and after six months at follow-up (T1). In addition, students in the intervention group complete a questionnaire after consultation with a YHC professional. The YHC professional likewise completes a questionnaire after each consultation with a student in the intervention group. To measure the level of medical absenteeism, data is retrieved from the registration system from school.

The level of medical absenteeism is obtained from the *absenteeism registration system* from the school in numbers of hours during a period of 12 weeks before T0 and T1; and also during the six months period between T0 and T1. Schools also provide information on the support that was given by the school to the student i.e. school meetings, social work or appointments with the psychologist.

The *baseline questionnaire* (T0) contains the following measurements; socio-demographic characteristics, behavioral determinants, mental health conditions, and overall wellbeing. Data on socio-demographic characteristics of the students will be gathered, by including questions regarding gender, age, educational level, country of birth, parents’ country of birth, and home environment [32, 33]. Behavioural determinants are assessed by questions about school career, physical activity, financial situation, sexual behaviour, smoking, alcohol use and drug abuse. In addition, absenteeism is measured by using the questionnaire based on the questionnaires developed by Municipal Public Health Services and health institutes [34]. Mental health conditions are assessed by using the Centre for Epidemiologic Studies Depression scale (CES-D) [35] and the 12-item Short Form Health Survey (SF-12) to measure health-related quality of life [36]. The CES-D scale is a 20-item scale used to determine the clinical relevance of depression. Selected items will cover the main components of depressive symptoms such as depressed mood, guilt, feelings of inferiority, feelings of helplessness, despair, loss of appetite, sleep and psychomotor retardation [37–39]. The SF-12 questionnaire is used to measure Quality of Life [36]. Six questions refer to the functional status, including physical and social functioning and physical and emotional limitations. Four questions describe well-being including mental health, vitality and pain. One question relates to the overall health condition. In addition, seven questions are included measuring the duration and incidence of medical absenteeism and truancy as well as the reasons for it.

The *questionnaire at follow up (T1)* is similar to the baseline questions except for the exclusion of questions on fixed socio-demographic variables and the addition of questions about the satisfaction with the YHC professional consult.

The *YHC consultation questionnaire for students* consists of 11 items assessing the extent to which the YHC professionals have worked in accordance with the MASS protocol (exposure to intervention). In addition, 10 questions are included measuring the students’ appreciation of the consultation with the YHC professional. Finally students are asked to rate the entire consultation on a scale from 1 to 10, with 1 being the most-negative evaluation score and 10 being the most-positive one.

The *YHC consultation questionnaire for the YHC professionals* includes items assessing the extent to which the school (*N* = 4) and the YHC professionals (*N* = 11) work in accordance with the MASS protocol. In addition, the Dutch version of the self-sufficiency matrix (SSM-D) [40, 41] is completed by the YHC professionals. The SSM-D addresses the ability of students to provide for themselves regarding 11 specific life-domains (e.g. income, daytime activities, housing, mental health) as assessed by the YHC professional. Each life domain of the SSM is measured by a single item. On each item, students are evaluated on 5-point Likert-type scales, from ‘in crisis’ (1) to ‘thriving’ (5). Finally, the perceived value of the MASS intervention according to the YHC professional including the biological model and the SSM-D, is assessed.

### Power considerations

Participating schools invite students who meet the inclusion criteria (i.e. reported sick four times in 12 weeks or more than six consecutive school days) to participate in the study. We will include 480 students, 240 in the control group and 240 in the intervention group. Allowing a loss-to-follow-up of 50% we assume to have complete data on 240 students (120 in the intervention and 120 in the control group). Taking into account cluster randomization with Intra Class Correlation of 0.10, assuming 15 participants per school class and an equal standard deviation (SD) of medical absenteeism in hours during a period of 12 weeks before T1 of 30 h [42], alpha 0.05 and a power of 0.80, in the case of one-sided testing, a difference in medical absenteeism of 15 h between Intervention and Control group at T1 can be established.

### Statistical analyses

In order to evaluate effect of the effect of the intervention the scores on outcomes of both groups measured during follow will be compared, adjusted for baseline findings. For continuous outcome measures such as duration of the medical absenteeism and psychometric test results (multiple) linear regression analyses will be applied. Dichotomous outcomes, such as progressing into the next year or obtaining a basic educational qualification degree, will be evaluated by using a multiple logistic regression analysis.

The research condition will be included as an independent variable. Baseline values of the outcomes are added to the model as covariates. As students are clustered within school locations multilevel analyses will be applied.

Potential moderation of intervention effects by gender, family situation, ethnicity, psychosocial and psychiatric problems (depression, drug abuse, ADHD) is explored by adding an interaction term to the regression models. Stratified analyses will be performed when significant interactions (*p* < 0.10) are observed.

Experiences of students and YHC professionals with the different elements of the MASS intervention as performed by YHC professionals will be evaluated by performing descriptive analyses.

## Discussion

Medical absenteeism is positively associated to early school dropout and may result in physical, mental, social and work-related problems later in life. The aim of the MASS intervention is to limit the medical absenteeism of students by arranging appropriate care, educational adjustments and adequate support [19, 21]. This paper describes the design of a controlled before-and-after study, which aims to evaluate the effectiveness of the MASS intervention as well as its contributing factors and interfering factors among intermediate vocational education school students.

It is hypothesized that implementation of the MASS intervention and incorporating a referral to a YHC professional is effective in terms of reducing medical absenteeism. Results of this study will show whether the YHC professional added value in the MASS intervention. Moreover, the study will provide insight in which factors contribute and/or moderate to the intervention’s effectiveness and may provide useful suggestions for improvement.

A strength of this study is that it will be conducted in the daily setting schools and YHC practice, which will facilitate future implementation. The participating schools and YHC professionals cover the urban and rural areas of the Netherlands, which will support the generalizability of our findings. Nevertheless, the schools and students are not randomized which can result in differences between the groups. We have tried to minimize this potential effect by correcting for potential confounders. Another strength is that we look at the extent to which the MASS intervention is applied by de YHC professional and gain insight into the care provided by the school to the individual students.

As the MASS intervention had already been implemented at the intervention schools, we could not capture strict criteria for referral to the YHC professional. The criteria for referral to a YHC professional were determined by each individual school which is in line with the MASS protocol, which may result in a variety of used criteria. On the other hand, this will give a realistic approach of the effects of the implementation of the MASS intervention. Also, the MASS-protocol requires great commitment of the schools to identify and refer students to YHC professionals [43]. It is therefore questionable how many students eventually will be identified and/or referred to the YHC professional.

In conclusion, the premise is that students with high rates of medical absenteeism receiving a customized reintegration plan and supervision from school and the YHC professional show a reduction in medical absence and easier return back to school compared to students receiving usual care. When MASS including the use of a YHC professional is effective, a comprehensive evaluation of full implementation of the MASS intervention at schools may follow.

## Abbreviations

CAT: Care Advisory Team
CES-D: Centre for Epidemiologic Studies Depression scale
CS: Control school
IS: Intervention school
MASS: Medical Advice for Sick-reported Students
SF-12: 12-item Short Form Health Survey
T0: Baseline questionnaire
T1: Follow – up questionnaire
YHC: Youth Health Care
